# Effects on keratinocytes of the traditional combination of herb extract (Royal Oji Complex) implicated the improvement of young children's skin moisture and barrier

**DOI:** 10.1111/srt.13682

**Published:** 2024-04-14

**Authors:** Thuy‐Tien Thi Trinh, Jae Hee Choi, Jee‐Eun Yang, Woon Ha Kim, Pham Ngoc Chien, Linh Thi Thuy Le, Nguyen Ngan‐Giang, Pham Thi Nga, Sun‐Young Nam, Chan‐Yeong Heo

**Affiliations:** ^1^ Department of Plastic and Reconstructive Surgery Seoul National University Bundang Hospital Seongnam Republic of Korea; ^2^ Zero to Seven Inc. Seoul Republic of Korea; ^3^ Korean Institute of Nonclinical Study Seongnam Republic of Korea; ^4^ Department of Biomedical Science College of Medicine Seoul National University Seoul Republic of Korea; ^5^ Department of Medical Device Development College of Medicine Seoul National University Seoul Republic of Korea

**Keywords:** claudin‐1 (CLD1), herb extract, hyaluronic acid synthase 3 (Has3), involucrin (IVL), loricrin (LOR), Royal Oji Complex, tissue‐isolated keratinocytes (TIKC)

## Abstract

**Background:**

Natural products are often friendly and can be used on children's skin after systematic and careful research. Therefore, in this study, the Royal Oji Complex (ROC), a product with natural ingredients, was used to study their effectiveness on keratinocytes taken from the skin of children from 0 to 3 years old.

**Method:**

Normal human epidermal keratinocytes and tissue‐isolated keratinocytes (TIKC) from young donors were treated with three different concentrations of ROC: 0.1, 1, and 10 ppm. The mRNA expression of the epidermal barrier's essential genes, such as *hyaluronic acid synthase 3 (Has3)*, *involucrin (IVL*), *loricrin (LOR)*, and *claudin‐1 (CLD1)* was investigated using qRT‐PCR. Ceramide content was measured by ELISA, with retinoic acid (R.A.) and amarogentin (AMA) serving as positive controls.

**Results:**

ROC significantly elevated *HAS3* gene expression in HEKn cells, especially at 10 ppm, indicating potential advantages for skin hydration in young infants. *IVL* increased at first but decreased as ROC concentrations increased. *LOR* was upregulated at lower ROC concentrations but reduced at higher doses. *CLD1* gene expression increased considerably in HEKn but reduced with increasing ROC doses. Ceramide concentration increased somewhat but not significantly at 10 ppm.

**Conclusion:**

ROC shows potential in altering keratinocyte gene expression, with unique responses in HEKn and TIKC from young donors. While changes in ceramide content were insignificant, these results help to comprehend ROC's multiple effects on young children's skin.

## 1 INTRODUCTION

The human skin is one of the body's most extensive and sophisticated organs, and it forms a physical barrier to the environment with an essential function in limiting passive water loss from the body, protecting the body against harmful agents, and managing the relationship between the body and the environment.^1^, ^2^, ^3^ The skin comprises multiple distinct layers with unique anatomical characteristics; each layer is characterized by the position, shape, morphology, and stage of differentiation of keratinocytes.^4^

The normal progression of keratinocyte differentiation assists in maintaining the integrity of the epidermis, which acts as a critical barrier restricting the organism from its environment.^5^ However, when an injury occurs, this normal self‐renewal of keratinocytes is disturbed, activating the wound‐healing process.^6^

Keratinocytes in the skin's granular layer produce keratohyalin granules, including the proteins profilaggrin, loricrin, and lipid‐enriched lamellar bodies. Loricrin (LOR), filaggrin (FLG), and involucrin (IVL) are the primary structural components of the cell envelop keratohyalin granules, which are cross‐linked by transglutaminase enzymes. Tight connections in the granular layer restrict the movement of solutes and water between keratinocytes (and hence loss to the environment) and play an essential role in epidermal barrier development. ^1^, ^7^, ^8^

Within the outermost layer of the skin, known as the stratum corneum (SC), FLG separates from keratins and breaks down into individual amino acids, facilitated by many proteases, such as caspase‐14, bleomycin hydrolase, and calpain‐1. This process generates the essential natural moisturizing factor (NMF), and it is crucial for controlling skin moisture, pH, protection against sunlight, and immune system regulation.^8^, ^9^, ^10^, ^11^, ^12^, ^13^, ^14^

The protein Claudin‐1 (CLD‐1) is another skin structure component, that creates tight junctions and is found in high levels in the skin's epithelium. When the expression level of claudin‐1 is reduced, it may disrupt the normal functioning of the epithelial barrier, which may result in the development of allergies in different areas of the body.^15^

Hyaluronic acid (HA), found in the epidermis, contributes to the formation of an effective barrier and the growth and specialization of keratinocytes. HA is produced by enzymes situated on the inner surface of the plasma membrane and is subsequently released as polymers directly into the extracellular matrix (ECM). Three HAS isoenzymes, namely HAS1, HAS2, and HAS3, have been found in mammals. Among these isoenzymes, HAS3 is very active and is responsible for synthesizing low molecular weight polymers; there is significant mRNA expression of HAS3 in healthy keratinocytes.^16^, ^17^

Epidermal ceramides, mainly accounted for by acyl‐ceramides, are crucial for skin barrier function. They are secreted from stratum granulosum cells to form a part of the cornified envelope and create sheets of lipid lamellae, resulting in an impenetrable barrier in the SC.^18^

Primary human keratinocyte cultures serve as a very effective model for investigating epidermal development since they accurately replicate the stages of epidermal cell differentiation.^19^ Keratinocyte culture systems have found extensive application in both biological and pharmacological research. Some of these culture systems have been specifically designed to facilitate high‐throughput screening of drugs aimed at treating psoriasis.^6^, ^20^, ^21^, ^22^

For instance, Qiao et al. examined the impact of vernix lipids (10 infant skin samples) on the expression of FLG, a precursor of the natural moisturizing factor and inflammatory indicators in normal human epidermal keratinocytes in vitro.^23^ This research emphasized the usefulness and applicability of the in vitro culture of the keratinocyte model for evaluating the bioactivity and effectiveness of novel pharmacological products.

For centuries, natural substances have been used to treat and support skin health. The interest in the health impacts of plants has recently risen owing to their safety and suitability in formulating medications and cosmetics.^24^ Familiar sources of natural components include herbs, fruits, flowers, leaves, minerals, water, and land. The impact of natural ingredients in skincare products relies on their effectiveness in in‐vitro and in‐vivo and the specific dermatological formulation in which they are included.^24^

Royal Oji complex (ROC) in the current study is a product composite from *Macadamia ternifolia* seed oil and five natural herbs (*Prunus mume* fruit extract, *Prunus persica* leaf extract, *Morus alba* leaf extract, *Salix alba* bark extract, and *Sophora japonica* root extract), which was developed based on *the journal of Hosancheong*.^25^ Moreover, the ROC is a bioactive compound like lipids and flavonoids produced by bioconversion technology using *Candida bombicola*. It is reported that this complex has biosurfactant characteristics, which could enhance skin hydration, improve the skin barrier, and reduce skin irritation. Therefore, we aim to evaluate the effect on several skin moisture and skin barrier biomarkers of ROC ex‐vivo using retinoic acid (R.A.) and amarogentin (AMA) as reference control reagents for inducing the keratinocytes in vitro and evaluate the expression of multiple genes in keratinocytes. All‐*trans* R.A., or Vitamin A, is required for cellular growth and differentiation, a ligand for nuclear R.A. receptor that binds to and upregulates the gene expression.^26^ Retinol is necessary for the proliferation and differentiation of keratinocytes in human skin.^27^, ^28^ Correspondingly, AMA is a secoiridoid glycoside with many biological functions that promote keratinocyte differentiation and influence cutaneous inflammation.^29^, ^30^

In the current study, we developed an in vitro model using keratinocytes isolated from skin biopsies or normal human primary epidermal keratinocyte cell line from the cell bank to investigate the effect of ROC on the expression of skin moisture (HAS3, FLG), and skin barrier biomarkers (IVL, LOR, CLD1, and Ceramide).

## 2 MATERIALS AND METHODS

### 2.1 Chemicals and reagents

ROC product was developed and obtained from ZOE BIO Inc. (Seoul, Korea), previously reported.^25^ R.A. (Vitamin A acid) and AMA were purchased from Sigma (St. Louis, MO, USA). Coating Matrix (type I collagen), Fetal Bovine Serum (FBS), and Trypsin‐EDTA for primary cells were obtained from Gibco‐Life Technologies™ (Carlsbad, CA, USA). Keratinocyte Growth Medium (KGM), Hank's Balanced Salt Solution (HBSS), Antibiotics/Antimycotics, and Collagenase/Dispase were purchased from Sigma (St. Louis, MO, USA).

### 2.2 Cell culture and treatment conditions

Normal human primary epidermal keratinocytes from Neonatal Foreskin (HEKn) cells were obtained from the American Type Culture Collection (Cat No. PSC‐200‐010) (Manassas, VA, USA) and cultured under the manufacturer's guideline. Briefly, HEKn cells were cultured in KGM at 37°C, 5% CO_2,_ and treated with different concentrations of ROC, R.A., and AMA at 37°C, 5% CO_2_ for 24 h. After the indicated incubation time, the cell pellets were collected to isolate of total mRNA or protein for further experiments.

### 2.3 Isolation and cultivation of primary human keratinocytes from human skin biopsies

Skin tissues were received as donations after circumcision surgeries from patients aged three or younger; the experiment procedure was reviewed and approved under IRB number H‐2303‐124‐1414. The human skin biopsies from patients under three were collected and used to isolate primary keratinocytes following previous reports.^31^

Briefly, the biopsied skin tissues were obtained to isolate the thin epidermis and the dense dermis, followed by incubation with antibiotics and Collagenase/Dispase in KGM medium for 12–16 h at 4°C. Afterward, samples were rotated on a rotator at room temperature for 15–20 min in Trypsin‐EDTA solution, passed through a 70‐µm mesh filter, and centrifuged at 200 g for 5 min. The cell pellets were collected and dissolved in KGM medium for counting the number of cells with a hemocytometer and seed on the pre‐coated Coating Matrix (type I collagen) plates. After stable cultivation of the isolated keratinocytes, cells were treated with different concentrations of ROC, R.A., and AMA for 24 h, at 37°C, 5% CO_2_. Cell pellets were collected after incubation to isolate total mRNA or protein for further analysis.

### 2.4 Total RNA isolation and real‐time quantitative reverse transcriptase polymerase chain reaction (qRT‐PCR)

Total RNA was extracted from the treated cells using RNAiso Plus (total RNA extraction reagent) (TaKaRa, Shiga, Japan) following the manufacturer's guidelines. Approximately 1 µg of total RNA was used for synthesizing cDNA by RevertAid First Strand cDNA Synthesis Kit from Thermo Scientific (Waltham, MA, USA). A TB Green® Premix Ex Taq™ II (TaKaRa) was used for quantitative PCR on a ViiA™ Real‐Time System (Thermo Scientific) under the following thermal cycles starting with denaturing at 95°C for 30 s, then 40 cycles of amplification at 95°C for 5 s and 60°C for 35 s. The relative mRNA expression was defined using the comparative C_T_ method using a reference gene, the 2^−∆∆Ct^ method as previously reported.^32^ 36B4 gene was chosen as the reference gene because of its convergence with a single‐copy gene. This gene shows minimal variability between persons; therefore, it can serve as an appropriate reference gene for assessing gene expression.^33^

### 2.5 Measurement of ceramide concentration

The keratinocyte cells were treated with R.A., AMA, or ROC at the indicated concentration and incubated for 24 hours at 37°C. The culture supernatant was collected to measure ceramide content using the Human Ceramide ELISA Kit (Cat No. MBS7254089) from MyBioSource (San Diego, CA, USA). Total extract protein concentration was determined using Pierce™ BCA Protein Assay kit (Cat No. 232225) from Thermo Scientific (Rockford, IL, USA).

### 2.6 Statistical analysis

The data were presented as the mean value ± the standard of the mean bar from three repeated independent experiments. All graphs were generated by Graph Prism 9 Software (Boston, MA, USA). Ceramide concentrations and mRNA expression data were analyzed in comparison with the no‐treatment group using the paired t‐test in the SPSS statistical program (IBM Corp., Armonk, NY, USA) and denoted as ^*^ *p *< 0.05, ^**^ *p *< 0.01, ^***^ *p *< 0.001.

## 3 RESULTS

### 3.1 Scheme and experiment setup for evaluating the effect on skin moisture biomarker and the skin barrier biomarker in keratinocytes induced by ROC

**FIGURE 1:**
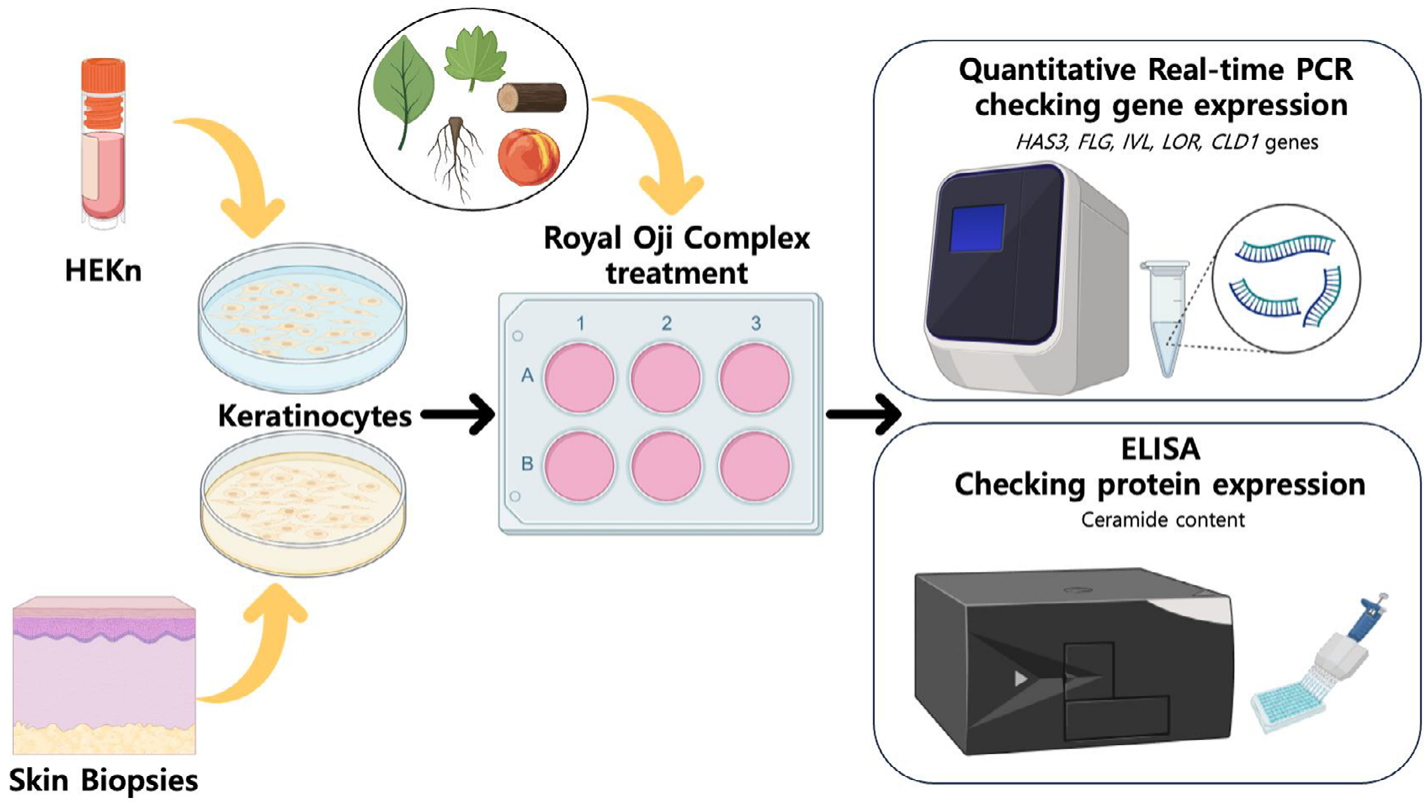
The scheme of an ex‐vivo experiment to assess the efficiency of the Royal Oji compound in inducing biomarkers of hydration and structure in keratinocytes.

To evaluate the effects of the ROC product, we cultured normal human primary epidermal keratinocytes from Neonatal Foreskin (HEKn) cells and keratinocytes isolated from patients under 3 years old. These cells were treated with the ROC product at different concentrations (0.1, 1, and 10 ppm), as depicted in Figure 1. After treatment, we collected the cells and used them to isolate total RNA and protein in order to assess the expression of several biomarkers associated with moisture improvement and barrier structure enhancement.

### 3.2 The skin moisture biomarkers induced by ROC

To determine the effect of the ROC on skin moisture improvement, we conducted experiments to evaluate whether the treatment of this complex could induce alterations in *HAS*3 and *FLG* gene expression in normal human epidermal keratinocytes (HEKn) or isolated keratinocytes from young donors.

After ROC treatment, the expression of the *hyaluronic acid synthase 3 (HAS3)* gene was significantly upregulated in HEKn cells, starting from the lowest ROC treatment concentration of 0.1 ppm. The expression of the *HAS3* gene was 1.82 times higher than in the untreated group.

Furthermore, this induction of the *HAS3* gene was in a concentration‐dependent manner, which increases with the evaluation of the ROC treatment concentration, and the highest expression level was observed at 10 ppm, showing a 4.36‐fold increase.

**FIGURE 2:**
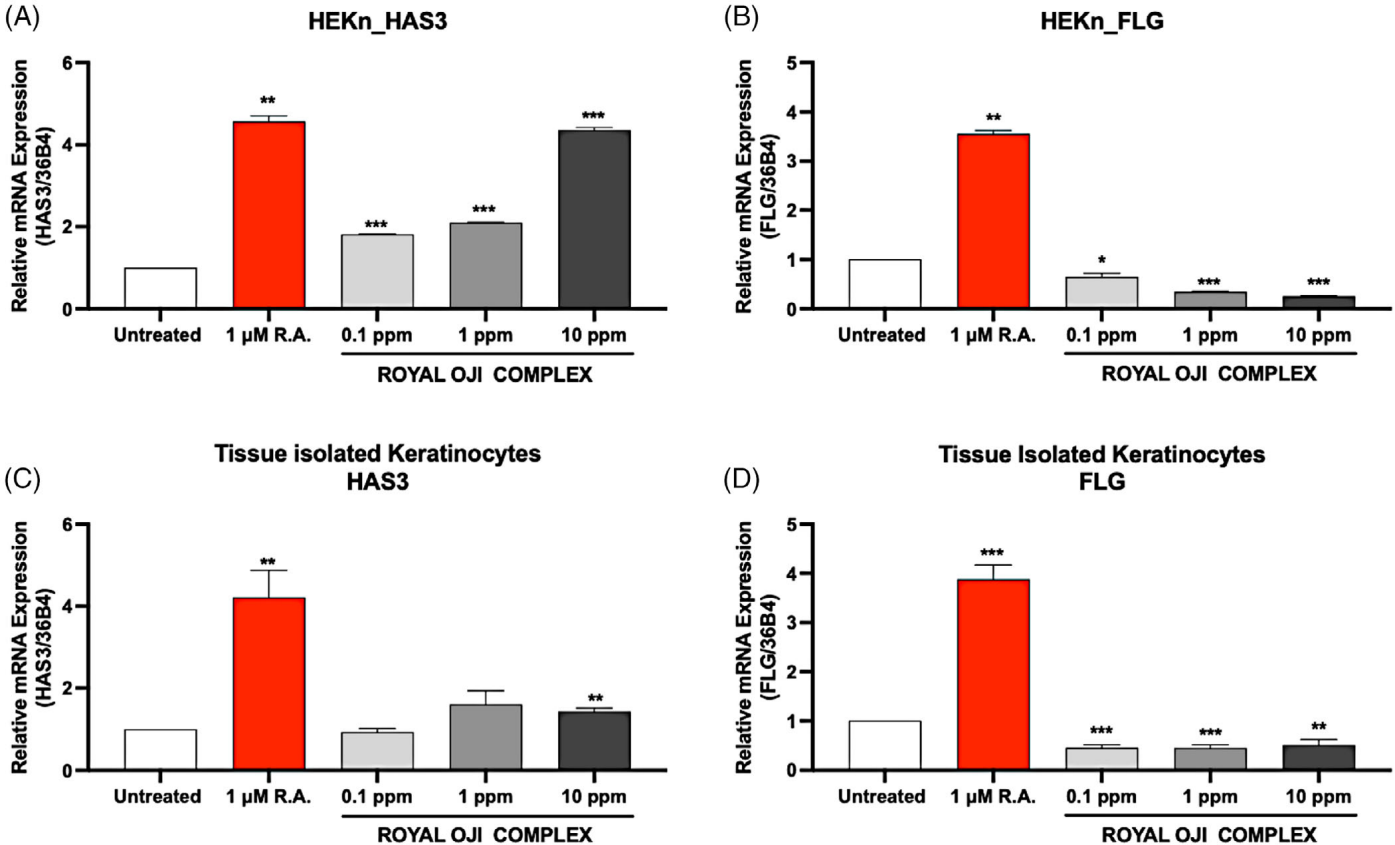
The effect of Royal Oji Complex (ROC) on the expression of the *HAS3* gene (A and C) and *FLG* gene (B and D) in HEKn and tissue‐isolated keratinocytes (TIKC), respectively. The results were obtained using the qRT‐PCR method for evaluating the mRNA expression from HEKn (*n* = 3) or TIKC (*n* = 9) after treatment with indicated concentration of ROC. ^*^ *p *< 0.05, ^**^ *p *< 0.01, ^***^ *p *< 0.001 compared with untreated control.

This highest dose of ROC treatment exhibited a comparable induction of the *HAS3* gene expression to that of the R.A. positive control treatment (4.57 folds). The relative mRNA expression level of the *HAS3* gene in HEKn cells is presented in Figure 2A.

When the tissue‐isolated keratinocytes (TIKC) were performed, the significant induction of the *HAS3* gene was observed in the highest ROC treatment concentration only (at 10 ppm); however, it was still lower than the R.A. positive control treatment. The data of *HAS3* gene expressed in TIKC was presented in Figure 2C.

In contrast, in responding to ROC treatment, the expression of the *FGL* gene was statistically significantly decreased in both HEKn and TIKC, which is reduced dose‐dependent. The downregulation of the *FGL* gene expression was observed from the lowest treatment concentration of ROC at 0.1 ppm with only 0.65 folds with HEKn; and 0.46 folds with TIKC.

In addition, the lowest expression of the *FGL* gene was observed at the highest treatment concentration at 10 ppm of ROC treatment with HEKn at only 0.26‐fold compared with the un‐treatment control (Figure 2B). Meanwhile, R.A. treatment control induced the *FGL* gene expression at 3.56 folds in HEKn cells and 3.88 folds in TIKC (Figure 2B,D).

### 3.3 Effect of ROC on skin barrier structure

To evaluate the effect of the ROC on skin barrier improvement, we conducted a qRT‐PCR experiment to investigate whether the treatment of this complex could regulate the gene expression of several skin barrier biomarkers such as IVL, LOR, and CLD1 in normal human epidermal keratinocytes (HEKn) or TIKC from young donors.

**FIGURE 3:**
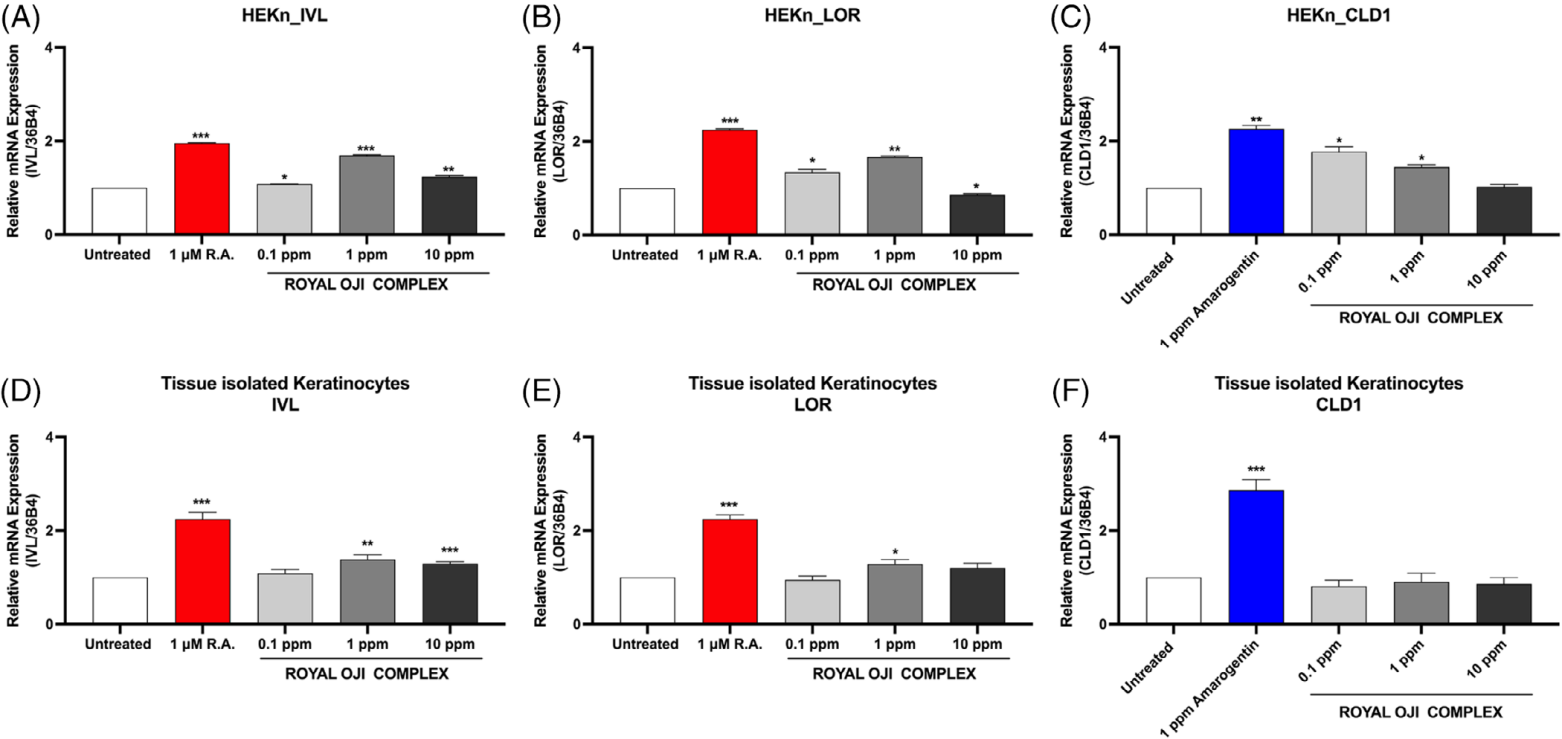
The effect of Royal Oji Complex (ROC) on the expression of *the Ivl* gene (A and D), *Lor* gene (B and E), and *Cld1* gene (C and F) in HEKn and tissue‐isolated keratinocytes (TIKC), respectively. The results were obtained using the qRT‐PCR method for evaluating the mRNA expression from HEKn (*n* = 3) or TIKC (*n* = 9) after treatment with indicated concentration of ROC. ^*^ *p *< 0.05, ^**^ *p *< 0.01, ^***^ *p *< 0.001 compared with untreated control.

The expression of the *IVL* gene was significantly upregulated in both HEKn and TIKC cells, in response to the ROC treatment at 1 ppm with an increase to 1.7 folds in HEKn and 1.39 folds in TIKC cells when compared with the untreated group. The relative mRNA expression level of the *IVL* gene in HEKn and TIKC cells is presented in Figure 3A,D.

Interestingly, this upregulation pattern slightly decreases at the highest concentration of ROC treatment of 10 ppm to 1.24 folds in HEKn and 1.29 folds in TIKC.

Furthermore, the ROC treatment could induce the *LOR* gene expression significantly, which increased the expression level of LOR, which started from 0.1 to 1 ppm in HEKn, showing a 1.34, and 1.66 folds increase, respectively. However, the highest dose of ROC treatment exhibited a slight down expression of the *LOR* gene to that of the un‐treatment control (0.86 folds), presented in Figure 3B.

Meanwhile, when the TIKC was used for incubating with ROC, a significant increase of the *LOR* gene expression was only observed at 1 ppm of ROC treatment, and it was lower than the R.A. positive control treatment. The data of the *LOR* gene expressed in TIKC was presented in Figure 3E.

In contrast, in responding to ROC treatment, the expression of the *CLD1* gene showed a statistically significant increase in HEKn only, which is, however, decreased with the increase of the ROC treatment concentration from 1.77, 1.45, and 1.03 folds at 0.1, 1, and 10 ppm, respectively. However, no significant difference in the *CLD1* gene expression was observed from the treatment of ROC in TIKC. The data on *CLD1* gene expression is presented in Figure 3C,F.

### 3.4 Effect of ROC on ceramide secretion level

Ceramide is a major component of lipids in the SC, accounting for 30%–40% of its content and responsible for water retention and skin barrier function.^34^, ^35^ Therefore, the ceramide content in keratinocytes could implicate better skin moisture and barrier function. To investigate this, we conducted experiments on HEKn and TIKC with varying concentrations of ROC (0.1, 1, 10 ppm) to assess its influence on ceramide expression.

After 24‐h incubation with ROC and control treatments of R.A., and AMA, the cell pellet was harvested, and homogenated to determine ceramide content using an ELISA assay.

**FIGURE 4:**
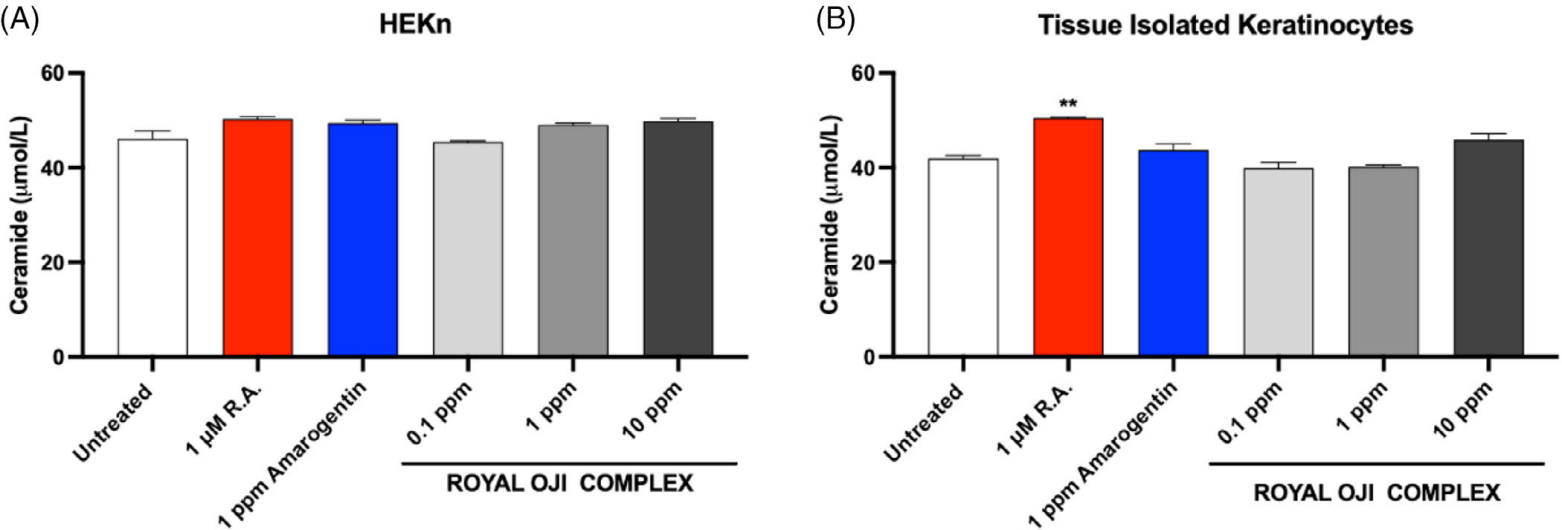
The effect of ROC on the secretion of ceramide from HEKn (*n* = 3) (A) or tissue isolated keratinocytes (TIKC) (*n* = 9) (B) after treatment with indicated concentration of ROC. ^*^ *p *< 0.05, ^**^ *p *< 0.01, ^***^ *p *< 0.001 compared with untreated control.

As shown in Figure 4, the ceramide concentrations of each treatment condition were not significantly different from untreated group, except for the R.A. treatment in TIKC. Although the average ceramide concentration in the ROC treatment at the highest concentration (10 ppm) showed a slight increase (49.84 for HEKn and 45.86 for TIKC), it was not statistically significantly different from the untreated group (46.06 for HEKn, and 41.93 for TIKC in the untreated group).

## 4 DISCUSSION

ROC in the current study is a product developed from five natural herbal extracts (*Prunus mume* fruit extract, *Prunus persica* leaf extract, *Morus alba* leaf extract, *Salix alba* bark extract, and *Sophora japonica* root extract) combined with *Macadamia ternifolia* seed oil and biological fermentation using *Candida bombicola* based on previous research.^25^

ROC contains *Sophora japonica* extracts with keratinocyte activation, proliferation, and differentiation‐regulating effects that prevent skin thickening and epithelial hyperplasia.^36^, ^37^

*Prunus mume* extract is another component of ROC that exhibits several physiological activities, antioxidant benefits, and anti‐melanogenesis properties.^38^, ^39^ Additionally, ROC contains *Prunus persica* leaf extract which has a unique acylated flavonol glycoside known for its high antioxidant capabilities, making it a promising anti‐inflammatory component in cosmetics.^40^ Furthermore, several studies have shown that *Prunus persica* leaf extract may protect keratinocytes and fibroblasts from cell damage caused by ultraviolet radiation.^41^ *Salix alba* bark and *Morus alba* leaf extract in ROC are recognized for their anti‐inflammatory and antioxidant qualities in natural medicine and cosmetics. *Salix alba* bark has notably enhanced antioxidant capacity and is safe for keratinocytes.^42^ The *Morus alba* leaf extract may decrease DNA damage and accelerate the S phase of the cell cycle in keratinocytes, as previously shown.^43^

Moreover, the bioconversion Oji complex, created using grafting bioconversion technology, has a more significant concentration of lipids, flavonoids, and amino acids of different sorts and has far more advantageous benefits on the skin. The biomarkers associated with skin hydration, skin barrier enhancement, and skin protection exhibit a dose‐dependent increase in HaCaT cells treated with Bioconversion Oji complex, reported recently by research from Lee et. al.^25^

In this study, our primary objective was to investigate the effect of the ROC on skin moisture and skin barrier improvement via inducing the related gene expressions in normal human epidermal keratinocytes (HEKn) and TIKC from the young donors (younger than 3‐year‐old donors). This study could elucidate the possible effects of ROC on baby skin health. In the current study, both HEKn and TIKC were affected by ROC treatment, which regulated epidermal hydrate genes (*Has3, Flg*), skin barrier‐related genes (*Ivl, Lor, Cld1*), and ceramide content.

Our results indicate that ROC treatment led to a significant upregulation of the *HAS3* gene expression in HEKn cells, particularly at the highest concentration of 10 ppm. This upregulation was comparable with the retinoic treatment as a control, indicating the potential of ROC to influence the expression of HA, which is associated with skin hydration. However, when TIKC was examined, the induction of *HAS3* gene expression was less pronounced and did not surpass the positive control. This suggests that the effect of ROC on gene expression may vary depending on cell resources. In particular, the difference in the resources of HEKn and TIKC is the potential of the genetic and phenotypic variability where the patient‐derived keratinocytes were isolated from different individuals who may have varied genetic backgrounds and health conditions and exhibit a broad spectrum of genetic and phenotypic variability.^44^, ^45^, ^46^ Meanwhile, HEKn are more homogenous and have been meticulously characterized, ensuring a consistent and predictable profile, and reducing variability in research outcomes.^47^, ^48^ The genetic and phenotypic variability of patient‐derived keratinocytes can offer a broader understanding of individual responses to ROC. At the same time, the uniformity of HEKn allows for establishing baseline effects of ROC on a more controlled genetic background.

In contrast to *HAS3*, the expression of the *FLG* gene was decreased in response to ROC treatment in both HEKn and TIKC, particularly at the highest ROC concentration. FLG is an essential component for barrier formation; its deficiency significantly impacts the epidermal barrier, affecting the organization of the keratin filaments of the cytoskeleton and the structure of the cornified envelope.^12^ The disorder of FLG gene expression was observed and recorded in several dermal diseases, particularly in those related to abnormalities of keratinization and the skin barrier, such as psoriasis,^49^ ichthyosis vulgaris,^50^ atopic dermatitis,^51^ etc.

Keratinocytes are the major constituent of epidermal cells, which could further differentiate to optimize the skin barrier by synthesizing keratohyalin granules composed of profilaggrin and loricrin. Moreover, they can also be denucleated to turn into confined cells at the uppermost keratinocytes in the granular layer.^52^ The cornified envelope comprises a mixture of cytoskeletal and barrier‐related molecules, such as LOR, FLG, filaggrin‐2 (FLG2), andIVL.^53^ These molecules are linked together by transglutaminase 1 and transglutaminases 3 and 5, and they also involve K1, K10, and desmosomal proteins (envoplakin and periplakin). All of these molecules play a crucial role in preserving the proper function of the barrier.^54^, ^55^, ^56^

The present study indicates a decrease in the expression of the FLG gene, especially when exposed to the highest concentration of ROC. This finding highlights the need to consider the concentration of ROC utilized carefully. However, it is notable that the decrease in FLG gene expression was more pronounced in HEKn cells compared to TIKC. Therefore, the differences in gene expression responses between cell resources merit further investigation. FLG is the main resource of several major components of NMF in the SC; therefore, the decrease in FLG expression by the effects of natural herbal extract such as ROC on keratinocytes may lead to the decrease of NMF.^11^, ^57^ Therefore, further investigation on NMF and FLG degrading enzymes must also be addressed. Moreover, the decrease in FLG expression may related to the suppression of the ROC in managing proliferation and inflammatory effects, particularly on keratinocytes.^54^, ^58^, ^59^

Furthermore, some cornified envelop precursor genes of the epidermal differentiation complex were also investigated, including IVL and LOR in this research.

Our results showed that ROC treatment led to a significant upregulation of the *IVL* and *LOR* gene expression in HEKn cells. However, the highest concentration of 10 ppm of ROC treatment showed a slight decrease in the expression of *IVL* and *LOR* genes in both HEKn and TIKC. This upregulation amplified the potential of ROC to influence the expression of IVL and LOR, which is associated with the epidermal structure. This regulation effect of ROC on these genes may vary depending on cell resources and not in a dose‐dependent pattern.

Our study also examined ceramide content, a vital component of the SC and an essential element for skin moisture and barrier function. While ROC treatment at the highest concentration showed a slight increase in ceramide content in both HEKn and TIKC, this increase was not statistically significant compared to the untreated group. Only the positive control treatment, R.A., exhibited a notable increase in ceramide content in TIKC. These findings suggest that ROC may have a limited impact on ceramide levels in the tested cells.

Recognizing the limitations of our work is crucial, particularly the need for more research to investigate the underlying processes accountable for the observed alterations in gene expression and ceramide levels. Moreover, the study's emphasis on in vitro tests may not comprehensively represent the intricacy of skin reactions in vivo. In current study, ROC seems to have less effect on the ceramide level in the conventional culturing method, however, in advanced cellular environment, such as the dimensionality of the cell culture, it can affect cellular responses and outcomes, including the metabolism and production of ceramides.^60^, ^61^ This study focuses primarily on ex vivo keratinocyte cultures, further combining data from studies on tissue‐engineered skin models and human skin equivalents may result in a more complete understanding of the effect of ROC. Besides, 2D and 3D culturing can be used for examining keratinocyte differentiation, advancing our understanding of skin biology under ROC treatment. By combining information from these models, more dependable conclusions may be drawn.

In summary, this research sheds light on the possible impacts of ROC on skin health, especially gene expression. While our data indicate that ROC affects gene expression patterns in keratinocytes, further study is needed to explain the processes at work and determine the effects of ROC in vivo. The outcomes of this study potentially serve as a blueprint for subsequent research endeavors in the realm of skin health.

## 5 CONCLUSIONS

Our research elucidates the in vitro effect of the ROC on genes responsible for moisture retention and structural integrity in keratinocytes. It offers an understanding of improving skin hydration and strengthening the skin's protective barrier when using ROC. Furthermore, we observed that the genes accountable for moisture regulation are activated depending on the dosage. However, the genes responsible for structural development do not exhibit the same activation pattern. Hence, our research has discovered the potential of using ROC as a skin barrier enhancer.

## Data Availability

The data that support the findings of this study are available from the corresponding author upon reasonable request.
